# A new option of reconstruction after extensive chest wall resection

**DOI:** 10.1016/j.clinsp.2025.100679

**Published:** 2025-05-15

**Authors:** João Paulo Cassiano de Macedo, Pedro Henrique Xavier Nabuco-de-Araujo, José Ribas M. de Campos, Paulo M. Pêgo-Fernandes, Ricardo M. Terra

**Affiliations:** a Thoracic Surgery Department, Instituto do Coração (InCor), Hospital das Clínicas da Faculdade de Medicina da Universidade de São Paulo (HCFMUSP), São Paulo, SP, Brazil; b Thoracic Surgery Department, Instituto do Câncer do Estado de São Paulo (ICESP), Hospital das Clínicas da Faculdade de Medicina da Universidade de São Paulo (HCFMUSP), São Paulo, SP, Brazil

**Keywords:** Sternal tumor, Chest wall tumor, Sternectomy, Chest wall resection, Reconstruction, Vitagraft®, Myocutaneous flap

## Abstract

**Objective:**

Chest wall reconstruction plays an important role in the outcomes of chest wall resection. However, there being a huge variety of materials at disposable, the best option has not yet been well defined. The Vitagraft® is a synthetic and absorbable material, that works as an osteoinduction and osteoconduction for bone regeneration. It consists of a β-phase Tricalcium Phosphate Ceramic (β-TCP) and the Polylactic Glycolic Acid Polymer (PLGA). Therefore, this study intends to assess the safety of Vitagraft® use in the chest wall.

**Methods:**

A prospective study, in which the patient’s chest was reconstructed with Vitagraft®. Each patient was followed after the procedure for at least three months. The following variables were considered: KPS, ECOG, preoperative treatment, defect size, myocutaneous flap, the time between surgeries, complications, and mortality.

**Results:**

Eight resections were performed due to tumor findings. One reconstruction was a treatment for sternal cleft, another for Poland’s syndrome, and finally as a consequence of late sternal dehiscence. Primary closure was performed in 63.6 % of the patients. Vitagraft® was used in association with mesh in eight cases. Reoperation was required in two cases, and prosthesis removal for one of them. Respiratory failure and major systemic complications were not evidenced.

**Conclusion:**

In the present study, the first to assess Vitagraft® in chest wall reconstruction, the second operation was necessary for 28 %, and removal was mandatory for 14 %. Until now, chest postoperative tomography has been showing good prosthesis biocompatibility. The authors need further details about the ossification time especially relating to the size of the resection.

## Highlights

• Chest wall reconstruction plays an important role in the outcomes of chest wall surgery. The ideal prosthesis characteristics are already established, even though, the best choice is not well decided.
• The authors propose a new prosthesis for chest wall reconstruction.
• This study is the first to assess patients with chest wall disease reconstructed with Vitagraft®. It is a synthetic, biocompatible, and absorbable material, which has never been found in thoracic surgery before.
• Finally, the use of Vitagraft® has been shown to have good prosthesis biocompatibility.

## Introduction

Extensive chest wall reconstruction is still a challenging situation in thoracic surgery. The surgical purpose can vary from cosmetic, infection, or oncological treatment. Resections due to cancer are frequently discussed in multidisciplinary teams, generally composed of oncologists and thoracic and plastic surgeons. The association between the thoracic team and plastic surgeon generally allows extensive resection and ensures sufficient free margin.^1^ Soft tissue coverage is the main contribution of plastic surgeons, per se, optimizing results.

On the other hand, the necessity of a prosthesis is decided by the thoracic surgeon. Anterolateral defects require prosthetic replacement in defects ≥ 5 cm in diameter or including ≥ 4 ribs.^2^ The posterior defects, even 10 cm in size, do not require reconstruction because of the scapula and shoulder girdle support.^2^ Care should be taken in defects lower than the fourth rib posteriorly to avoid trapped scapula.

Le Roux and Sherma^3^ have already punctuated that the ideal prosthesis should be (I) Rigidity to abolish paradoxical movement; (II) Inertness to allow in-growth of fibrous tissue and decrease the likelihood of infection; (III) Malleability to fashion to the appropriate shape at the time of operation; and (IV) Radiolucency to ensure a better follow up. Unfortunately, the perfect match is still not found. New options for reconstruction need to be considered, despite the indication for prosthetic replacement still the same for years. The Vitagraft® is now a promising alternative. Consisting of a synthetic, biocompatible, absorbable, non-cytotoxic, non-immunogenic, and non-pyrogenic, it works as an osteoinductor and osteoconductor, for bone regeneration. Composed of a nanometric ceramic of Tricalcium Phosphate in the β-phase (β-TCP) and the copolymer Polylactic Glycolic Acid (PLGA). Although Vitagraft® is frequently seen in orthopedic scenarios, there is no evidence of chest wall surgery before. So, this study intends to assess the safety of use in extensive chest wall reconstruction.

## Methods

A prospective ongoing study in which patients were submitted to extensive chest wall resection and reconstruction based on Vitagraft®. This study was approved by the Ethics Committee (EC) of the institution where the work was carried out, under the number 56091721.6.0000.0068. All patients had to fill out the informed consent before the procedure. Inclusion criteria: chest wall resection no matter the purpose, oncological or palliative treatment. Exclusion criteria: chest wall resection due to infectious disease, patients without clinical conditions to surgical procedure, loss of follow-up. Each case was followed for a minimum period of three months after the surgical intervention. The following variables will be considered: KPS, histological type, previous local surgical treatment, the time between surgeries, defect size, type of myocutaneous flap, and mortality.

## Results

**Fig. 1:**
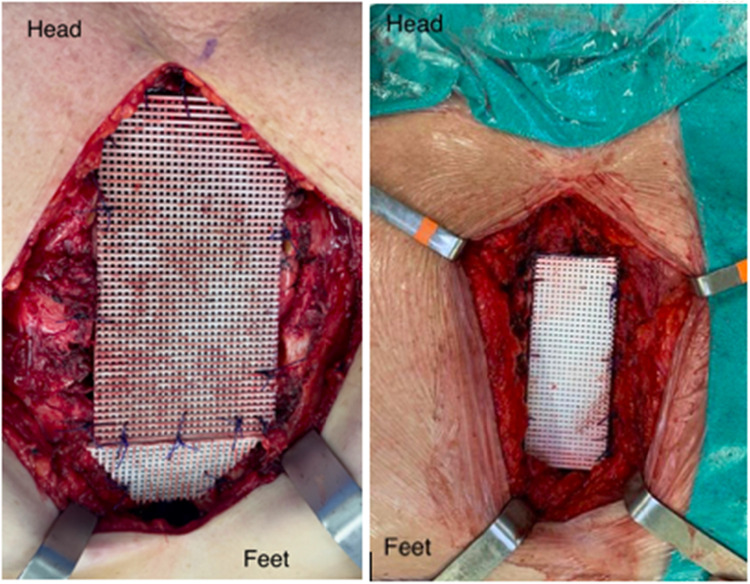
Vitagraft® tailored made after two distinct sub-total sternectomies.

**Fig. 2:**
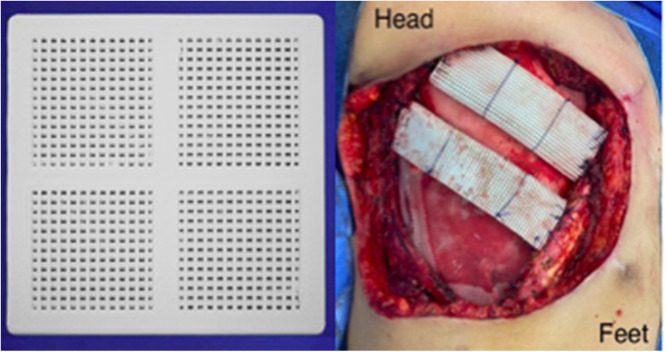
First the Vitagraft® presentation used in chest wall reconstruction. Second the two Vitagraft® ribs in association with PPM.

Demographic DATA was summarized in Table 1. From the total of eleven patients, eight resections were performed due to tumor findings, Table 2, Table 3. One patient was submitted to surgery as a consequence of a sternal cleft, and another because of Poland’s syndrome. One sternectomy was a consequence of late sternal dehiscence Table 2 and Fig. 1. Primary closure was evidenced in 63.6 % of the patients, while *Latissimus dorsi* was necessary once, and abdominal flaps were necessary in two cases, Table 4. Vitagraft® was used in association with polypropylene mesh (PPM) a total of eight times, Fig. 2. The Vitagraft® without association was used in two patients and once with bovine pericardium. Reoperation was required in two cases, and prosthesis removal was necessary for one of them. Paradoxical chest movement, respiratory failure, bleeding intractable chest pain, and major systemic complications were not evidenced. The area of defect size was calculated by multiplying the bottom-to-top measure by the side-to-side measure. The median was 60 cm^2^, varying from 7.5 cm^2^ to 324 cm^2^.

**Table 1:** Demographic DATA.

KPS^a^	Median 100
Age	**Median 46**
Sex	Female 8 (73 %)
	Male 3 (27 %)
Smoking	2 (18 %)
Diabetes	2 (18 %)
Obesity	2 (18 %)
COPD^b^	1 (0.09 %)
Hypertension	1 (0.09 %)
Alcoholism	1 (0.09 %)
CAD^c^	1 (0.09 %)
Coronary disease	1 (0.09 %)

a Karrnofsky.

b Chronic Obstructive Pulmonary Disease.

c Chronic Artery Disease.

**Table 2:** Reconstruction and main indication.

Reconstruction indication	Number (%)
Tumor	8 (72.7 %)
Sternal cleft	1 (0.09 %)
Poland’s syndrome	1 (0.09 %)
Sternal dehiscence	1 (0.09 %)
Total	11 (100 %)

**Table 3:** Tumor Types.

Tumor	Number (%)
Chondrosarcoma	3 (37.5 %)
Breast cancer	1 (12.5 %)
Phyllodes	1 (12.5 %)
Chondroma	1 (12.5 %)
Fibrous dysplasia/Multiple Exostosis	2 (25 %)
Total	8(100 %)

**Table 4:** Types of muscle flaps and Vitagraft® use.

Reconstruction (closure)	Number (%)
*Primary closure*	7 (63.6 %)
*Latissimus dorsi*	1 (0.09 %)
Abdominal flap	3 (27 %)
Vitagraft® use	**Number (%)**
Polypropylene mesh	8 (72.7 %)
Lonely	2 (18 %)
Bovine pericardium	1 (0.09 %)
Total	11 (100 %)

## Discussion

The safety assessment was necessary even because Vitagraft® is already used in orthopedics, and neurosurgery but not in chest wall reconstruction. The present study is the first to evaluate Vitagraft® application regarding extensive chest wall reconstruction. No major complications were evidenced either during surgery or the following period. Local complications were noticed twice.

**Fig. 3:**
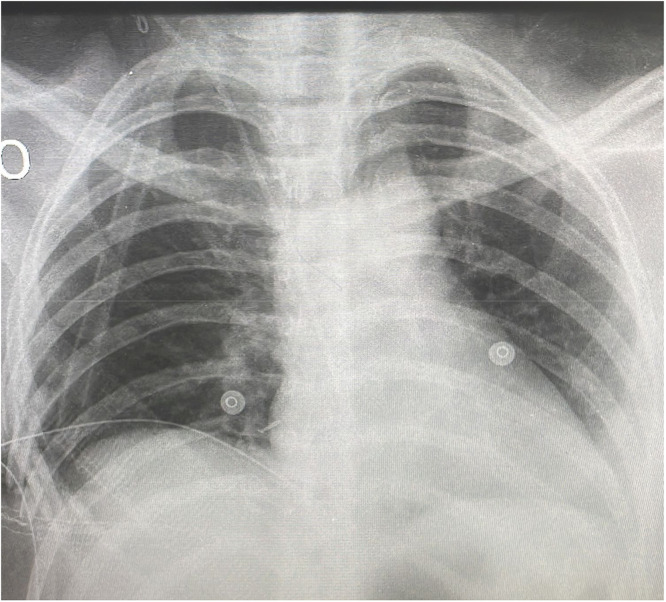
Chest X-Ray showing prosthesis radiolucency.

Local complications are frequently seen in chest resections. Bad outcomes are reported to occur in 37 % to 46 %^4^ of patients. Aghajanzadeh, M^5^ reported wound complications such as infection, dehiscence, flap loss, and hematoma in 8 % to 20 %. Chest X-ray was good at showing prosthesis radiolucency, Fig. 3, but computed tomography (CT) should be the primary imaging modality for the assessment of the local complications. Imaging has information about the prosthesis and potential identification of fluid and air accumulation adjacent to the prosthesis, which is indicative of deeper wound infection or associated pleural empyema.^6^ In addition, respiratory complications such as pneumonia, atelectasis, and respiratory failure are reported in 18 %, 20 % to 24 %^5^ of cases as a consequence of a flail segment of the chest wall. Respiratory problems were not noticed in our experience due to the absence of paradoxical chest movement. Besides, wound complications requiring a second operation were necessary for 28 %, and removal was mandatory for 14 %. The management of wound infections should be tailored to the severity of the infection (imaging clinical aspect presentation), and underlying disease. The first case was not associated with mesh. The female patient with previous breast reconstruction, in which the silicon prosthesis was damaged before the chest resection. The late operative wound remained producing fluid and the tomography showed only a partial ossification of the prosthesis. The authors do believe that all of these factors contributed to the redo surgery. The second complication was reconstructed in association with mesh and appeared to be a consequence of soft tissue cover, mainly a compound of fatty tissue despite muscle and a lack of alternatives for prosthesis presentation. In this case, the material was much less solid and the reconstruction was performed with a single Vitagraft® rib. The material was fractured and removed during the follow-up period. The authors suggested some changes after this episode to increase strength. Now it maintains the same ticking, but the structure is reinforced by some parallel solid bars. In addition, the authors now reconstruct the defect at least with two Vitagraft® ribs, not one as used to be.

Comparisons between different types of prostheses are frequently seen. In some cases, the prosthetic material used depends on the surgeon’s preference and experience.^7^ Deschamps C^8^ showed no significant difference in the postoperative outcome or complications between prolene mesh and polytetrafluoroethylene (PTFE) soft tissue patch for chest wall reconstruction. Weyant M^4^ compared rigid (marlex mesh associated with methylmethacrylate sandwich(MMM) to non-rigid reconstruction consisting either of polypropylene(PPM) alone or expanded PTFE. There was no significant difference in respiratory complications among prosthetic groups, but MMM prostheses had a significantly higher number of wound complications.

**Fig. 4:**
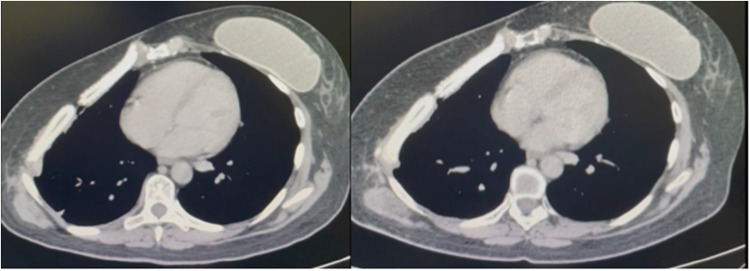
CT-Scan showing the reconstruction after three- and seven-months respectability.

Until now, the three-month postoperative chest tomography has been showing good prosthesis biocompatibility, Fig. 4, and safe use. The wound complication rate is similar to the literature but not related to respiratory failure. The use of this new prosthesis does not seem to increase inflammatory reaction as a consequence of reabsorption and ossification as time goes by. The authors need to increase the sample size and provide further details about the ossification time, especially relating to the size of the resection. The authors continue to collect information for further publications when the study is over.

## Authors disclosure and conflicts of interest

This work was supported by DMC equipment. The industry is responsible for Vitagraft® development.

## Note

Artificial intelligence was not used as a tool in this study.

## Conflicts of interest

The authors declare no conflicts of interest.
